# The predictive value of prostate spherical volume ratio in lower urinary tract symptoms and clinical progression of benign prostatic hyperplasia: a retrospective cohort study

**DOI:** 10.1007/s11255-024-04355-4

**Published:** 2025-01-15

**Authors:** Tangrao Ji, Kewei Huang, Biming He, Haifeng Wang

**Affiliations:** 1 Department of Urology, Shanghai East Hospital, School of Medicine, Tongji University, No. 150 Jimo Road, Pudong New District, Shanghai, 200120 China; 2 Department of Urology, Lanxi People’s Hospital, Jinhua, China; 3 School of Data Science, Fudan University, Shanghai, China

**Keywords:** Benign prostatic hyperplasia, Lower urinary tract symptoms, Magnetic resonance imaging, Clinical progression of benign prostatic hyperplasia

## Abstract

**Purpose:**

To evaluate the predictive value of Prostate Spherical Volume Ratio for Lower urinary tract symptoms and clinical progression of Benign prostatic hyperplasia. And compared with other prostatic anatomical parameters.

**Methods:**

A total of 154 patients with Benign prostatic hyperplasia who underwent MRI and urodynamics were included in the study, while prostate anatomical parameters such as prostate spherical volume ratio, prostate volume, intravesical prostatic protrusion, prostatic urethral length and presumed circle area ratio were determined based on MRI measurements. Average length of follow-up was 12 months. The primary outcome was follow-up for the clinical progression of Benign prostatic hyperplasia, defined as an increase in the International Prostate Symptom Score of at least 4 points, Benign prostatic hyperplasia-related prostatic surgery. Univariate and multivariate linear regression analyses were conducted to identify risk factors for lower urinary tract symptoms. Logistic regression analyses were conducted to identify risk factors for clinical progression of Benign prostatic hyperplasia.

**Results:**

Multivariate linear regression highlighted the significant association of prostate spherical volume ratio with lower urinary tract symptoms. In multivariable logistic regression analysis prostate spherical volume ratio is an independent risk factor for the clinical progression of Benign prostatic hyperplasia to an increase in the International Prostate Symptom Score of at least 4 points (OR = 1.53 *p* = 0.016) and Benign prostatic hyperplasia -related prostatic surgery (OR = 5.15 *p* = 0.020).

**Conclusion:**

Prostate spherical volume ratio has been significantly correlated with Lower urinary tract symptoms, and it was an independent risk factor for the clinical progression of Benign prostatic hyperplasia.

**This trial is registered on ClinicalTrials.gov:**

NCT06448533

**Supplementary Information:**

The online version contains supplementary material available at 10.1007/s11255-024-04355-4.

## Introduction

Benign prostatic hyperplasia (BPH) is one of the most common urological disorders in men aged over 50 years [1] and it is a progressive disease [2]. Lower urinary tract symptoms (LUTS) secondary to BPH are a significant concern in elderly men [3]. It was once widely believed that the size of the prostate gland, as indicated by its volume (PV), was correlated with the severity of LUTS. However, this conviction has recently been challenged [4–6]. Various anatomical parameters of the prostate, including intravesical prostatic protrusion (IPP), prostatic urethral length (PUL), prostatic urethral angle (PUA), bladder neck angle (BNA), and prostatic apex shape, have been identified as risk factors for LUTS in many studies [7–10]; however, it is difficult to explain the cause of LUTS in terms of a single parameter, and some of these parameters are still controversial [11].

Some theories have sought to devise more precise methods for predicting the degree of LUTS.

by leveraging calculations of prostatic anatomical parameters. One such theory is the presumed circle area ratio (PCAR), first proposed by Hiroki Watanabe in 1979 [12]. PCAR is defined as the ratio between the surface area of the prostate’s largest transverse section and the area of a hypothetical circle with an equivalent circumference to that of the aforementioned transverse section [12–14]. Hiroki Watanabe posits that as enlargement of the gland of the prostate, the shape of its transverse section transitions from triangular to circular, ultimately resulting in a more pronounced manifestation of LUTS due to urethral compression [12–14].

Most PCAR studies, including those by Watanabe, currently rely on two-dimensional measurements obtained through transrectal ultrasound [12–17]. However, the use of transrectal ultrasound for prostate measurement has the potential to deform the prostate due to the pressure exerted by the ultrasound probe [18], thereby influencing measurement accuracy. MRI of the prostate yields high-resolution tissue imaging [19], without inducing deformation of the gland due to compression. Furthermore, the three-dimensional reconstruction of the prostate morphology, derived from layer-by-layer scanning of prostate images, allows for the integration of a broader range of prostate parameter information. Consequently, this approach holds promise in enhancing the accuracy of predicting the severity of LUTS.

We hypothesized that a nearly spherical three-dimensional morphology of the prostate may be associated with more severe LUTS. Consequently, we introduced the concept of prostate spherical volume ratio (PSVR). This metric is defined as the ratio of the prostate’s volume, calculated through mathematical modeling based on all cross-sectional images obtained from prostate magnetic resonance (MR), to the volume of a sphere with the same surface area as the prostate.

To evaluate our hypothesis and explore the risk factors for the clinical progression of BPH, this study conducted a thorough investigation into the interrelation between PSVR and the severity of LUTS, ascertained through the measurement of maximum urinary flow rate (Qmax) and International Prostate Symptom Score (IPSS). We also assessed the predictive value of PSVR for the clinical progression of BPH, using BPH-related prostatic surgery and an increase in IPSS greater than or equal to 4 points as outcome measures, with a 12-month follow-up. Additionally, we compared these findings with those obtained from various other prostatic anatomical parameters.

## Materials and methods

### Patients

This study received approval from the ethics committee at Shanghai East Hospital and was conducted in accordance with the ethical guidelines specified in the Helsinki Declaration. A total of 231 males with BPH who underwent MRI, uroflowmetry, and had patient-report IPSS available at our department between September 2020 and April 2023 were included. Among these, 77 were excluded due to missing data (*n* = 26), previous prostate or urethral surgery (*n* = 30), prostate cancer (*n* = 10), and undergoing alpha-blocker therapy in the past month (*n* = 11). The final analysis focused on the remaining 154 patients. Patient characteristics included age, prostate-specific antigen (PSA), PV, IPP, PUL, PCAR, PSVR, Qmax, and IPSS.

### international prostate symptom score (IPSS) and urinary flow rate (Qmax)

IPSS is a well-recognized seven-item patient-reported questionnaire with a total score ranging from 0 to 35, where a higher score indicates more severe LUTS. Mild LUTS was defined as an IPSS of 0–7; moderate LUTS as 8–19 IPSS; and severe LUTS as 20–35 IPSS. Qmax is a widely used urinary flow measure in LUTS studies, with a lower value indicating more severe LUTS.

### Prostatic anatomical parameters

**Fig. 1:**
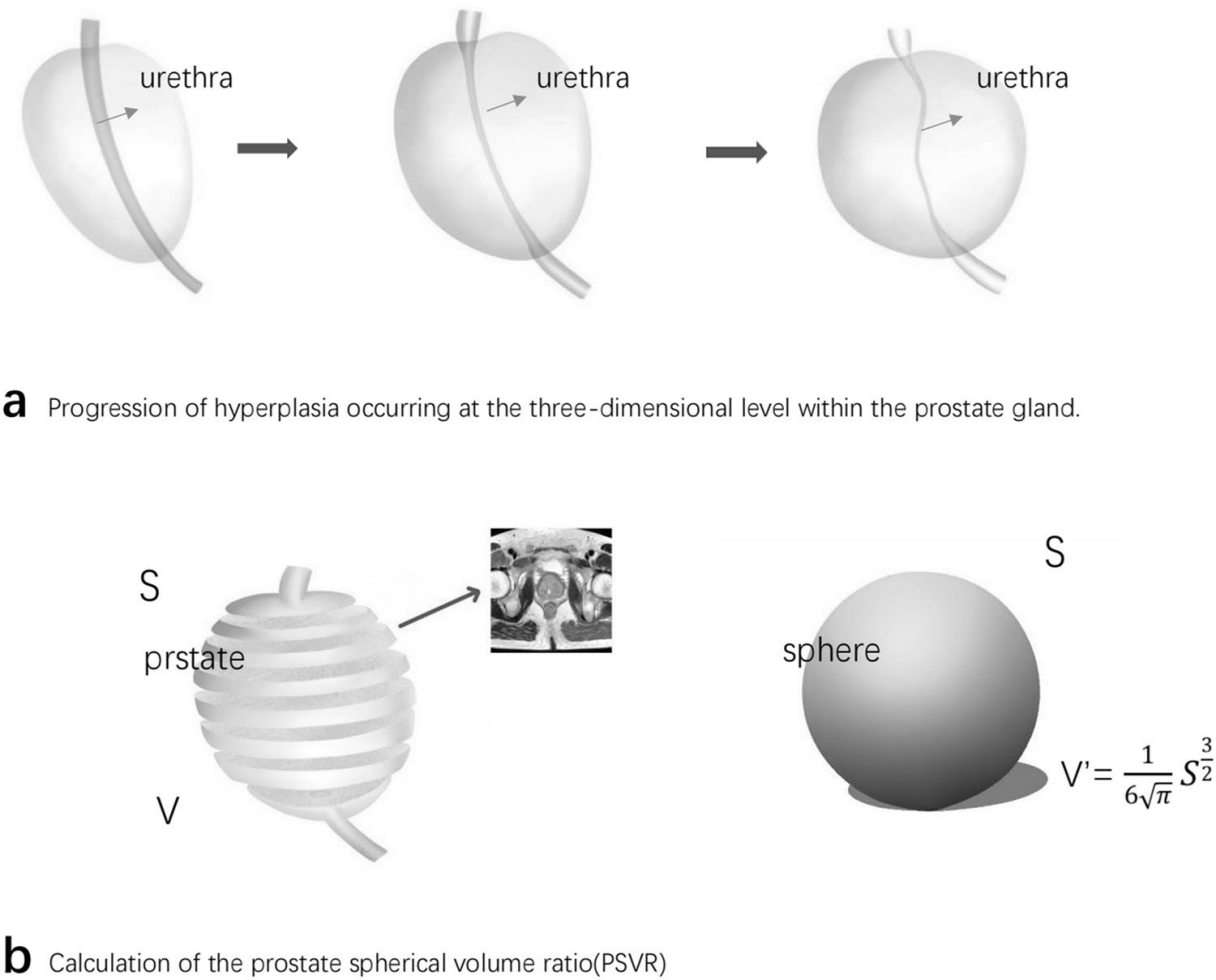
Progression of hyperplasia occurring at the three-dimensional level within the prostate gland and Calculation of the prostate spherical volume ratio(PSVR) **a** shows that with prostatic hyperplasia, the prostate morphology approximates a spherical shape and the urethra is compressed and thinned and delayed, leading to LUTS. **b** We constructed the prostate morphology from the magnetic resonance T2-weighted cross-sectional images by three-dimensional modeling and calculated the surface area (S) and volume (V) of the prostate.The volume (V') was calculated from the sphere with the same surface area (S).According to the equation of surface area and volume of a sphere $V^{\prime } = \frac{1}{{6\sqrt {\uppi } }}{{S^{3/2} }}$ PSVR = V/V^’^

All anatomical parameters were measured using T2-weighted images in MRI (DISCOVERY MR 750W, General Electric Company). PSVR was defined as the ratio of prostate volume and was developed as follows: First, we delineated the shape of the prostate by outlining the boundary of the prostrate in each transverse T2-weighted image. Second, following the concept of the Marching Cubes algorithm [20], we reconstructed the prostate’s shape at all depicted levels into a polyhedron and calculated the volume (V) and surface area (S) of the prostate. Third, the volume of the sphere is maximised under the condition that the surface area is fixed [21]. We calculate the volume of the sphere using the formula relating the surface area and volume of a sphere. Finally, we computed PSVR based on the V/V' formula. Thus, PSVR can reflect the similarity between the 3D morphology of the prostate and that of the sphere (Fig. 1). The PSVR value ranged from 0 to 1, with higher scores indicating a closer resemblance to the sphere.

**Fig. 2:**
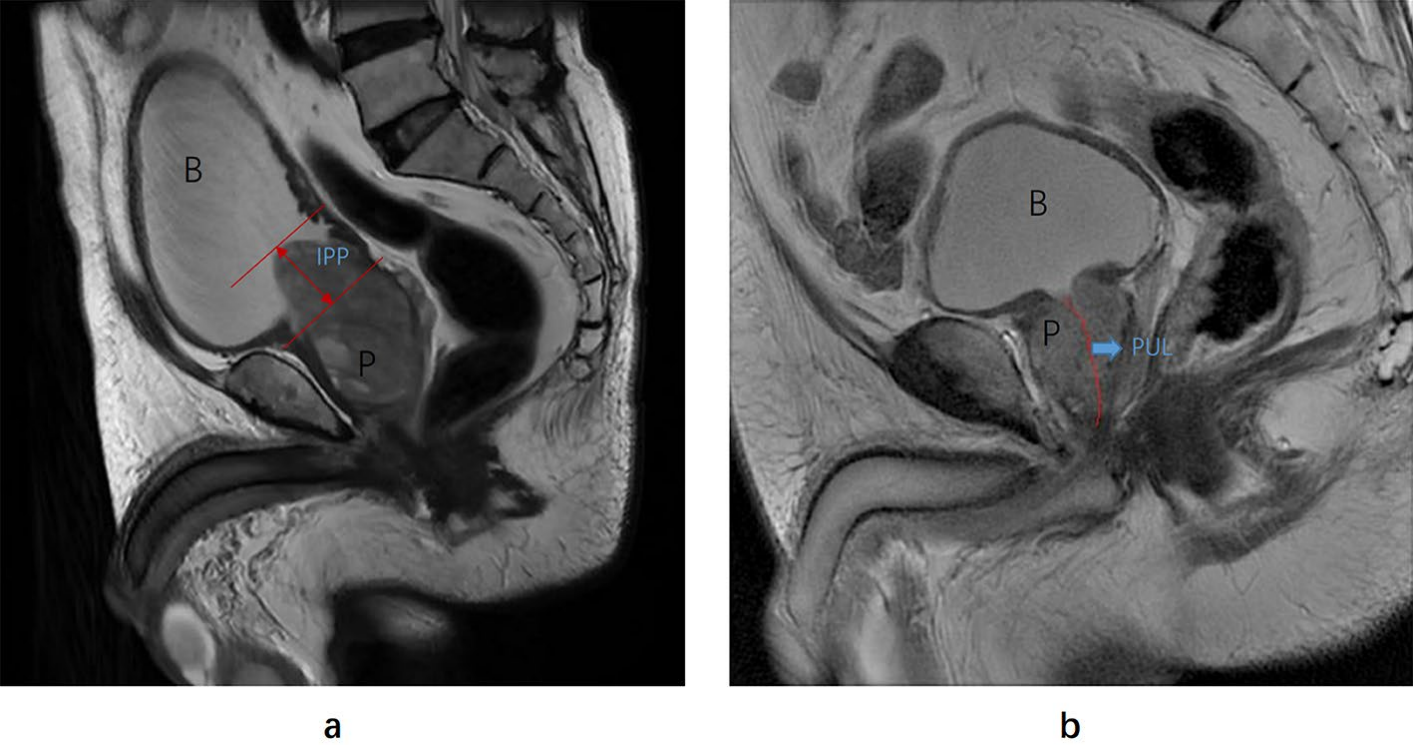
The measurement of the intravesical prostatic protrusion (IPP) and prostatic urethral length (PUL) on T2-weighted sagittal magnetic resonance imaging: **a** IPP is the vertical length from the protrusion of the prostate (P) to the base of the bladder **B**. The red line represents the IPP measurement. This patient is 72 years old with an IPP of 25.3 mm. **b** PUL is the length from the base of the prostate (P) to the apex of the prostate in midsagittal on MRI. The red line represents the PUL measurement. This patient is 68 years old with a PUL of 4.5 cm

PCAR refers to the ratio of the maximum transverse area of the prostate to the area of a hypothetical circle with the same circumference [12–14]. In this study, we selected the T2-weighted transverse section of the prostate with the maximum cross-sectional area, and calculated its area (S) and circumference (L) using the Open Source Computer Vision Library in Python. The area of the hypothetical circle with the same circumference was calculated as S' = L^2^/4π. Therefore, according to the definition of PCAR, PCAR = 4πS/L^2^. PV represents the prostate volume, which we calculated during prostate modeling. IPP is the vertical length from the protrusion of the prostate to the base of the bladder, first described by Chia [22], and can be measured using sagittal plane MRI [23]. PUL is the length from the base of the prostate to the apex of the prostate in midsagittal plane MRI [24]. Based on the methodology described above, we utilized T2-weighted sagittal images to measure the IPP and PUL, as illustrated in Fig. 2. It’s important to note that these prostatic anatomical parameters were measured based on MRI rather than ultrasound imaging. All researchers responsible for measuring or calculating these parameters were blinded to other clinical data.

### Outcomes

The primary outcome, described as the clinical progression of BPH, was the first occurrence of an increase in the IPSS of at least 4 points [25], BPH-related prostatic surgery. Patient follow-ups.

commenced at the point of enrollment, with subsequent visits occurring every three months. Follow-up session included the administration of the IPSS questionnaire and an assessment of surgical intervention. A total of 12 months of follow-up were conducted, or until the appearance of outcome indicators, whichever transpired first.

### Statistical analysis

In baseline characteristics, categorical variables were reported as the mean ± standard deviation (SD). Pearson’s product-moment correlation test was used to determine the relationships between prostate anatomical variables and voiding parameters, such as changes in the IPSS and Qmax.

In the linear analysis, first, univariate linear regression analyses were conducted to investigate the association of prostatic anatomical parameters with LUTS (based on the IPSS and Qmax). Second, prostatic anatomical parameters with *p* < 0.05 in the univariate analyses were further included in the multivariate linear regression analysis to identify independent predictors of LUTS.

Multivariate logistic regression analysis was used to identify independent predictors of clinical progression of BPH. Receiver operating characteristic(ROC) curves were constructed to assess the diagnostic accuracy of PV, IPP, PUL, PCAR and PSVR for the clinical progression of BPH. All reported p-values were two-sided, and statistical significance was set at p < 0.05. Statistical analyses were performed using SPSS 25 for Windows (IBM Corporation, USA).

## Results

A total of 154 patients diagnosed with BPH were included in the current analysis. Table 1 presents the characteristics of the entire study population. The subjects had a mean age of 66.8 ± 7.4 years, a height of 170.6 ± 4.7 cm, a weight of 70.9 ± 9.1 kg, a BMI of 24.3 ± 3.0, a PSA of 7.5 ± 10.9 ng/ml, an IPSS of 15 ± 8, a Qmax of 11.4 ± 7.2 ml/s, a PV of 40.1 ± 27.7 ml, an IPP of 11.1 ± 6.5 mm, a PUL of 5.2 ± 0.9 cm, a PCAR of 0.83 ± 0.04, and a PSVR of 0.74 ± 0.04. At 12 month follow-up, 74(48.1%) patients underwent BPH-related prostatic surgery, while among the 58 (37.6%) patients who did not undergo surgery, 10 (17%) experienced an increase in IPSS greater than or equal to 4 point. 3 patients were diagnosed with prostate cancer, and 19 patients were lost to follow-up.

**Table 1:** Clinic characteristic of the patients include in the study

Variable	Mean ± SD
Age(years)	66.8 ± 7.4
Height(cm)	170.6 ± 4.7
Weight(kg)	70.9 ± 9.1
BMI	24.3 ± 3.0
PSA(ng/ml)	7.5 ± 10.9
IPSS	15 ± 8
Qmax(ml/s)	11.4 ± 7.2
PV(ml)	40.1 ± 27.7
IPP(mm)	11.1 ± 6.5
PUL(cm)	5.2 ± 0.9
PCAR	0.83 ± 0.04
PSVR	0.74 ± 0.04

*BMI* Body mass index, *PSA* Prostatic specific antigen, *IPSS* International prostate symptom score, *Qmax* Maximum flow rate, *PV* Prostate Volume, *IPP* Intravesical prostatic protrusion, *PUL* Prostatic urethral length, *PCAR* Presumed circle area ratio, *PSVR* Prostate spherical volume ratio

Pearson’s correlation analysis revealed that PV (*r* = 0.189, *P* = 0.019), IPP (*r* = 0.433, *P* < 0.001), PUL (*r* = 0.245,  = 0.003), PCAR (*r* = 0.362, *P* < 0.001) and PSVR (*r* = 0.308, *P* < 0.001* P*) increased with increasing IPSS (Figure S1). In univariate and multivariate linear regression analysis, we evaluated the relationships between IPSS and potential variables, including Age, Height, Weight, BMI, PSA, PV, IPP, PUL, PCAR, and PSVR. Multivariate analysis showed that IPP [B:0.428, 95% confidence interval: (0.221, 0.635), p < 0.001] and PSVR [B:38.109, 95% confidence interval: (3.926, 72.292), p = 0.029] were the two most significant parameters associated with IPSS (Table 2).

**Table 2:** Results of univariate and multivariate linear analyses of the factors associated with total IPSS

Total IPSS	Univariate		Multivariable	
	B(95% CI)	P	B(95% CI)	P
Age	0.105(−0.061to0.271)	0.215		
Height	−0.094(−0.364 to 0.177)	0.494		
Weight	−0.010(−0.146 to 0.127)	0.891		
BMI	0.031(−0.389 to 0.450)	0.885		
PSA	0.048(−0.078 to 0.173)	0.455		
PV	0.052(0.009 to 0.096)	0.019	−0.036(−0.093 to 0.021)	0.218
IPP	0.468(0.301 to 0.635)	< 0.001	0.428(0.221 to 0.635)	< 0.001
PUL	2.008(0.709 to 3.306)	0.003	0.219(−1.519 to 1.958)	0.803
PCAR	71.056(41.743 to 100.378)	< 0.001	24.893(−9.836 to 59.623)	0.158
PSVR	61.33(30.976 to91.684)	< 0.001	31.109(3.926 to 72.292)	0.029

*IPSS* International prostate symptom score, *BMI* Body mass index, *PSA* Prostatic specific antigen, *PV* Prostate volume, *IPP* Intravesical prostatic protrusion, *PUL* Prostatic urethral length, *PCAR* Presumed circle area ratio, *PSVR* Prostate spherical volume ratio

Pearson’s correlation analysis showed that PV (*r* = −0.203, P = 0.012), IPP (*r* = −0.462, *P* < 0.001), PUL (*r* = −0.312, *P* < 0.001), PCAR (*r* = −0.293, *P* < 0.001), and PSVR (*r* = −0.391, *P* < 0.001) increased with declining Qmax (Figure S2). In univariate linear regression analyses, PV, IPP, PUL, PCAR, and PSVR were negatively associated with Qmax, but age, height, weight, BMI, and PSA were not. PV [B:0.067, 95% confidence interval: (0.019, 0.115), *p* = 0.007], IPP [B:−0.432, 95% confidence interval: (−0.603, −0.262), *p* < 0.001] and PSVR [B:−53.510, 95% confidence interval: (−80.898, −26.122), *p* < 0.001] were significant parameters associated with Qmax in multivariate linear regression analysis (Table 3).

**Table 3:** Results of univariate and multivariate linear analyses of the factors associated with Qmax

Qmax	Univariate		Multivariable	
	B(95% CI)	P	B(95% CI)	P
Age	−0.122(−0.261 to 0.016)	0.083		
Height	0.094(-0.034 to 0.393)	0.099		
Weight	0.030(−0.079 to 0.140)	0.548		
BMI	−0.025(−0.361 to 0.312)	0.886		
PSA	−0.027(−0.126 to 0.071)	0.584		
PV	−0.049(−0.087 to−0.011)	0.012	0.067(0.019 to 0.115)	0.007
IPP	−0.398(−0.529 to−0.266)	< 0.001	−0.432(−0.603 to −0.262)	< 0.001
PUL	−2.267(−3.404 to−1.131)	0.003	−0.620(−2.009 to 0.769)	0.378
PCAR	−47.725(−72.878 to−22.572)	< 0.001	3.076(−24.489 to 30.642)	0.826
PSVR	−64.696(−89.256 to−40.136)	< 0.001	−53.510(−80.898 to −26.122)	< 0.001

*Qmax* Maximum flow rate, *BMI* Body mass index, *PSA* Prostatic specific antigen, *PV* Prostate volume, *IPP* Intravesical prostatic protrusion, *PUL* Prostatic urethral length, *PCAR* Presumed circle area ratio, *PSVR* Prostate spherical volume ratio

In univariate logistic regression analyses, PV, IPP, PUL, PCAR, and PSVR were statistically correlated with BPH-related prostatic surgery, but only PSVR was statistically significant with BPH-related prostatic surgery(OR = 5.153 P = 0.02) in multivariate logistic regression. (Table 4). In univariable and multivariate logistic regression analysis, only PSVR remained statistically significant with an increase in the IPSS of at least 4 points, the p-values were 0.041 and 0.016. (Table 5).

**Table 4:** Univariable and multivariable logistic regression between anatomical parameters and BPH-related prostatic surgery at 12 months

Factor	Odd Ratio(95% CI)	p
Univariable		
PV	1.029 (1.011–1.047)	0.001
IPP	1.142 (1.063–1.226)	< 0.001
PUL	2.448 (1.513–3.960)	< 0.001
PCAR*10	5.234 (1.883–14.551)	0.002
PSVR*10	10.245 (3.400–30.877)	< 0.001
Multivariable		
PV	0.996(0.974–1.018)	0.697
IPP	1.071(0.983–1.165)	0.115
PUL	1.750(0.867–3.532)	0.119
PCAR*10	1.308(0.347–4.925)	0.692
PSVR*10	5.153(1.294–20.515)	0.02

*PV* Prostate valume, *IPP* Intravesical prostatic protrusion, *PUL* Prostatic urethral length, *PCAR* Presumed circle area ratio, *PSVR* Prostate spherical volume ratio, PCAR*10 Multiply the value of PCAR by 10, PSVR*10 Multiply the value of PCAR by 10

**Table 5:** Univariable and multivariable logistic regression between anatomical parameters and BPH-related prostatic surgery at 12 months

Factor	Odd Ratio(95% CI)	p
Univariable		
PV	0.983 (0.945–1.022)	0.381
IPP	0.459 (0.836–1.084)	0.459
PUL	0.747 (0.264–2.118)	0.584
PCAR*10	1.290 (0.282–5.901)	0.742
PSVR*10	1.271 (1.009–1.601)	0.041
Multivariable		
PV	0.948(0.863–1.042)	0.266
IPP	1.027(0.856–1.233)	0.774
PUL	0.978(0.107–8.951)	0.984
PCAR*10	0.550(0.068–4.454)	0.575
PSVR*10	1.539(1.085–2.185)	0.016

*PV* Prostate valume, *IPP* Intravesical prostatic protrusion, *PUL* Prostatic urethral length, *PCAR* Presumed circle area ratio, *PSVR* Prostate spherical volume ratio, PCAR*10 Multiply the value of PCAR by 10, PSVR*10 Multiply the value of PCAR by 10

**Fig. 3:**
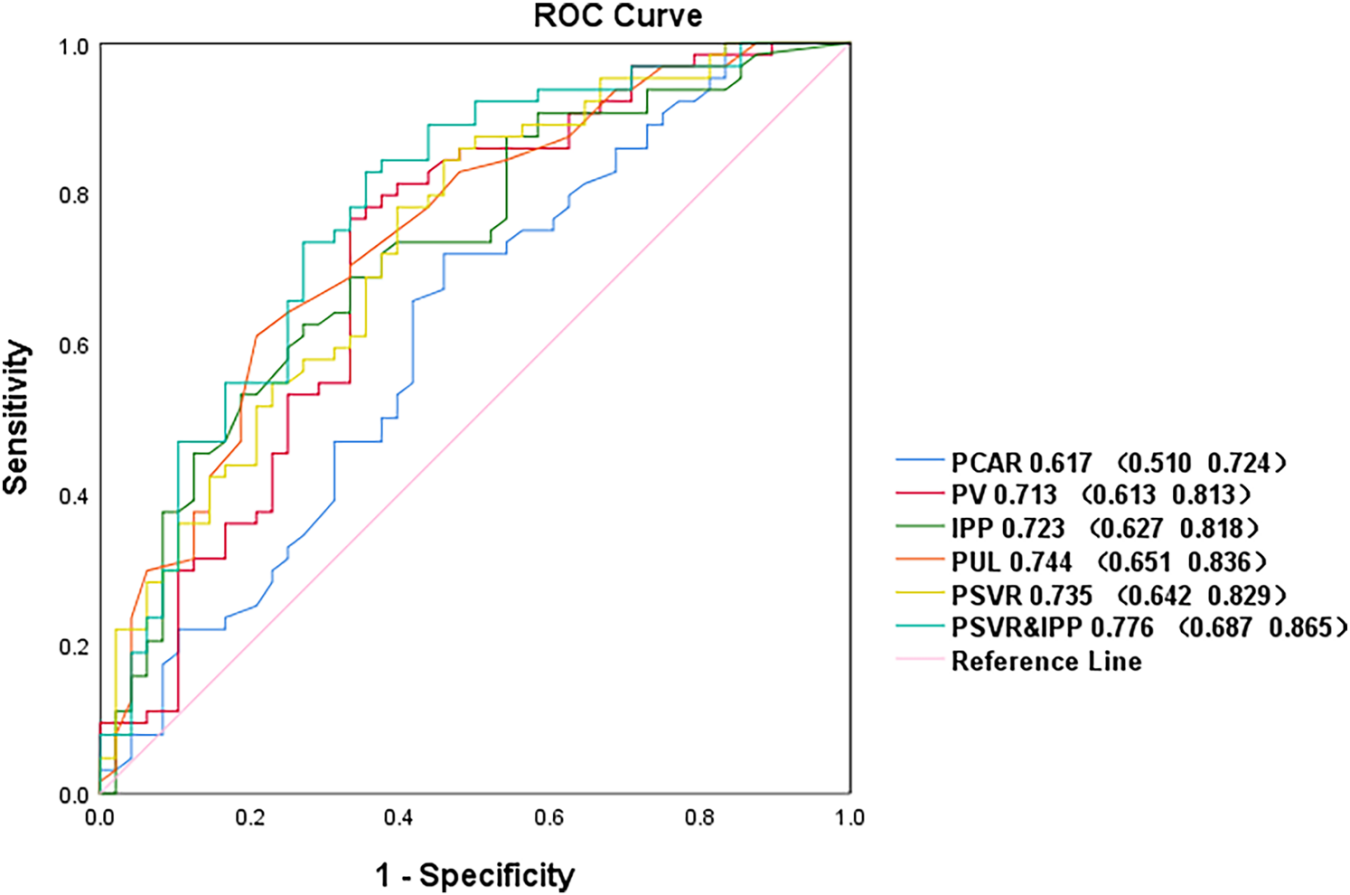
Receiver operating characteristics of PV, IPP, PUL, PCAR, PSVR and PSVR&IPP with BPH-related prostatic surgery. *PV* Prostate volume, *IPP* Intravesical prostatic protrusion, *PUL* Prostatic urethral length, *PCAR* Presumed circle area ratio, *PSVR* Prostate spherical volume ratio, *PSVR&IPP* PSVR combined IPP

To assess the predictive power of prostate anatomical parameters for surgery. We compared the area under the curve(AUCs) between PV, IPP, PUL, PCAR,PSVR and PSVR combined IPP( PSVR&IPP)with BPH-related prostatic surgery in the present analysis(*N* = 132).AUCs ranged from 0.617 to 0.744 (PCAR, 0.617; PV, 0.713; IPP, 0.723; PSVR, 0.735; PUL, 0.744; PSVR&IPP, 0.776) Fig. 3.

**Fig. 4:**
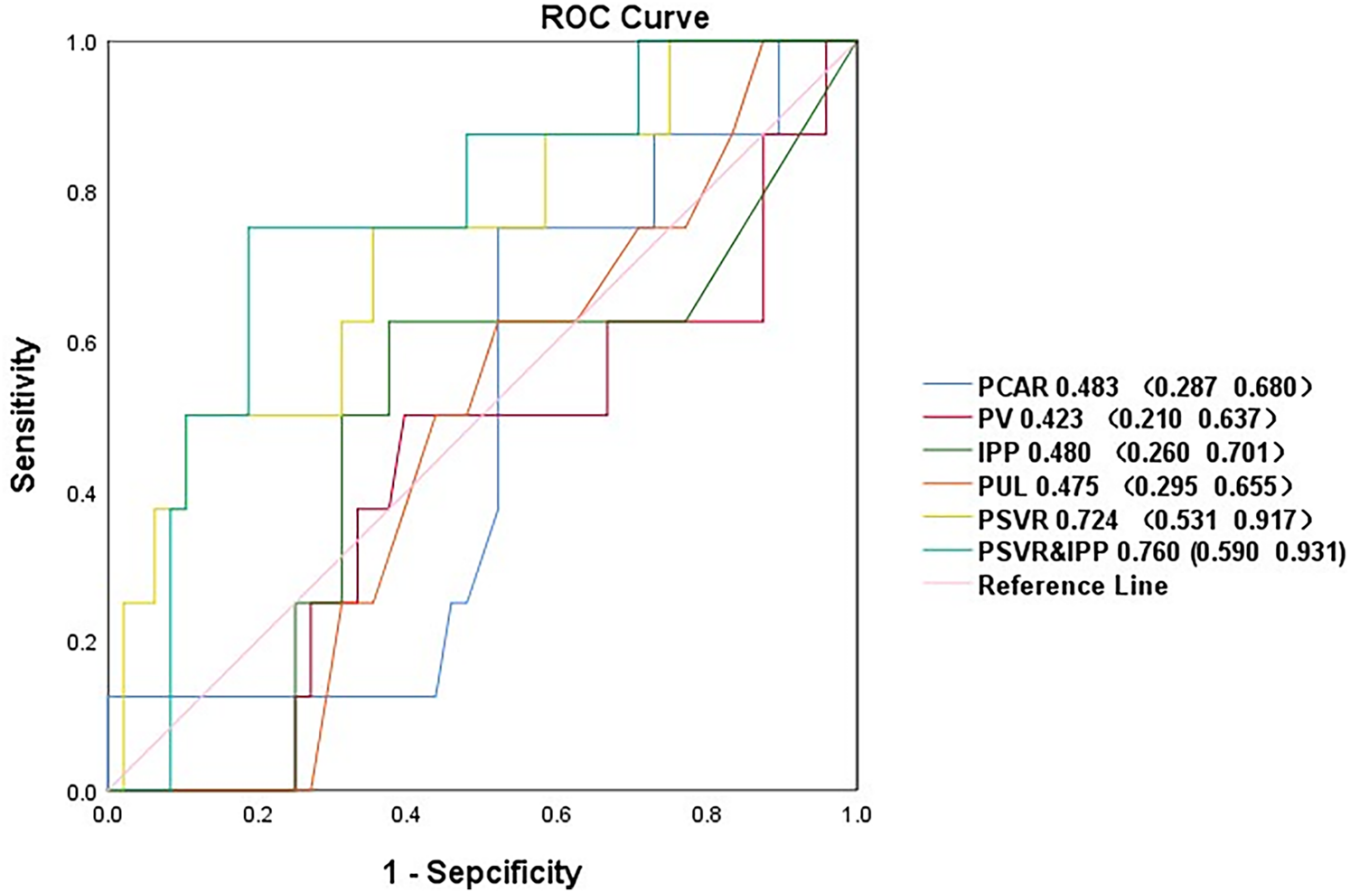
Receiver operating characteristics of PV, IPP, PUL, PCAR, PSVR and PSVR&IPP with an increase in the IPSS of at least 4 points. *PV* Prostate volume, *IPP* Intravesical prostatic protrusion, *PUL* Prostatic urethral length, *PCAR* Presumed circle area ratio, *PSVR* Prostate spherical volume ratio, *PSVR&IPP* PSVR combined IPP

To assess the predictive power of prostate anatomical parameters for increased IPSS. We also compared AUCs between PV, IPP, PUL, PCAR, PSVR and PSVR combined IPP( PSVR&IPP) with an increase in the IPSS of at least 4 points in the present analysis(*N* = 58).AUCs ranged from 0.423 to 0.724 (PV, 0.423; IPP, 0.480; PUL, 0.475; PCAR, 0.0.483; PSVR, 0.724; PSVR&IPP, 0.760) Fig. 4.

## Discussion

In this retrospective study, we evaluated the correlation between PSVR and other prostatic anatomical parameters with the degree of LUTS, and assessed the predictive value of PSVR and other prostatic anatomical parameters for the clinical progression of BPH. Our findings indicated that a higher PSVR or IPP was significantly associated with more severe LUTS. In multivariable logistic regression analysis PSVR is an independent risk factor for an increase in the IPSS of at least 4 points (OR = 1.53 p = 0.016) and BPH-related prostatic surgery (OR = 5.15 p = 0.020). The PSVR AUCs were 0.735 and 0.724 for the predicted diagnostic efficacy for BPH-related prostatic surgery and an increase in the IPSS of at least 4 points, respectively.

Benign prostatic hyperplasia (BPH) has long been a focus of research among urologists because of its high prevalence, high disease burden [3], and continuous progression. Various anatomical parameters of the prostate, including PV, IPP, PUA, BNA, have been identified as risk factors contributing to LUTS [7–10]. However, PV and PUA are controversial in some studies[4.5.6.11]. The application of IPP may also be limited because some patients with prostatic hyperplasia do not have prostatic glands protruding into the bladder. These individual prostate anatomical parameters may not provide a comprehensive assessment of LUTS. In our clinical practice, we observed that many patients with severe voiding symptoms often exhibited spherical prostate shape in MRI. Based on this observation, we hypothesized that the closer the prostate shape is to a sphere, the more severe the voiding symptoms. We therefore proposed the concept of prostate spherical volume ratio(PSVR). To test this hypothesis, we outlined the prostate’s shape at different MRI levels, used mathematical modeling to measure its surface area (S) and volume (V), and calculated the PSVR based on the ratio of V to the spherical volume (V’) with the same surface area (S). Our findings confirmed our hypothesis, showing a positive correlation between PSVR and IPSS and a negative correlation with Qmax.

Interestingly, as early as 1979, Japanese urologist Hiroki Watanabe proposed PCAR [12], a concept similar to our PSVR. They observed that the normal prostate appears triangular in transrectal ultrasound images, while early-stage hyperplasia takes on a crescent shape, progressing to a rounded shape in the later stages. Watanabe attributed this transformation to the prostate’s composition, encompassing the transitional and peripheral zones. As the prostate adenoma undergoes hyperplasia, the enlarged gland exerts pressure on the peripheral zone, causing it to protrude outward. As glandular hyperplasia progresses slowly over time, uniform pressure on the peripheral zone leads to compression and gradual rounding, eventually forming a rounded prostate shape. According to mathematical principles, the area enclosed by a circle with the same circumference is maximized, hence the more advanced the prostate hyperplasia, the closer it resembles a circle shape. Watanabe described and demonstrated this phenomenon using two-dimensional data. Consistent with their theory, our study also identified a positive correlation between PCAR and IPSS, along with a negative correlation with Qmax, further supporting this concept.

While both our proposed PSVR and PCAR perspectives focus on prostate shape, a key distinction lies between them: PSVR is three-dimensional (3D) whereas PCAR is two-dimensional (2D). In theory, PSVR offers a more comprehensive and realistic reflection of the prostate’s actual state compared to PCAR. This is due to PSVR’s ability to leverage a broader range of information from prostate images. Furthermore, most current studies on PCAR are based on measurements obtained through transrectal ultrasound [12–17]. These measurements entail several inherent shortcomings: they tend to underestimate actual prostate volume [26], they are heavily dependent on the proficiency of the ultrasound technician, and they are prone to inter-operator variability, leading to potential discrepancies in the values ascertained by various physicians, including the determination of the maximum cross-sectional area. Additionally, transrectal ultrasound requires inserting the ultrasound probe into the patient's body, which can introduce deformation due to compression of the prostate by the probe [18], thus increasing measurement errors. The use of MRI provides excellent resolution for examining prostate tissue, enabling visualization of the anatomical structures in the prostate region and facilitating precise measurements [19]. Therefore, our measurements for both PSVR and PCAR are based on multi-parametric magnetic resonance imaging. In our current study, our findings further support this theory. We observed that PSVR exhibited a stronger correlation with Qmax than PCAR (*r* = −0.391 vs. *r* = −0.293, *p* < 0.001). When combined with mathematical modeling, these advanced imaging techniques can more accurately and completely depict the prostate’s shape. BPH is a progressive disease, and the prediction of clinical progression of the prostate is of great value for both diagnosis and treatment of the disease. Although many scholars have investigated the relationship between PCAR, IPP, PUL, PV and luts, fewer studies have been conducted on the above parameters of BPH clinical progression.

Therefore, to explore risk factors for clinical prostate progression, we performed a 12-month follow-up analysis including the above prostate anatomical parameters and PSVR, and analysed their association with LUTS alongside their association with BPH clinical progression. All of these were positively correlated with IPSS and negatively correlated with Qmax, with IPP demonstrating the strongest correlation. These findings align with previous studies, such as Wang’s research [27], which established a positive correlation between PV and IPSS and a negative correlation with Qmax. Similarly, Kim [24] discovered that IPSS was higher in cases of small volume and long PUL compared to large volume and short PUL (*p* = 0.042), while Eze BU [28] found a positive correlation between IPP and IPSS (*r* = 0.406, *p* < 0.001), among other studies. In multivariate logistic regression analyses, PSVR was an independent risk factor for clinical prostate progression. It is worth noting that most prior studies relied on ultrasound images for data collection, whereas our study used magnetic resonance images. As a result, there may be slight variations in data measurement accuracy between the two modalities.

Several limitations must be acknowledged in this study: First, all measurements and calculations of prostate parameters were based on MRI, which helps mitigate errors associated with deformation compression in transrectal ultrasound measurements and reduces the reliance on ultrasound practitioners’ proficiency. However, the accuracy of prostate parameter measurements, particularly the establishment of PSVR, is contingent on the number of cross-sectional layers included in the MRI. Due to time constraints, the number of layers scanned was limited, resulting in a certain degree of deviation. Second, magnetic resonance imaging (MRI) incurs a greater financial burden in contrast to ultrasound techniques. Third, this was a retrospective study, and therefore, it unavoidably inherits the limitations associated with such study designs. Fourth, the sample size of this study was relatively small, and the data were derived from a single institute. Thus, further validation of the findings in other centers is warranted.

## Conclusion

PSVR, a novel concept introduced in this study, builds upon PCAR, serving as a metric to assess the degree to which the three-dimensional morphology of the hyperplastic prostate approximates a sphere. Our findings strongly suggest that PSVR is a better predictor of BPH clinical progression and LUTS. Recent technological advancements have made it increasingly feasible to seamlessly integrate 3D modeling with MRI measurements. This development holds promise for the application of PSVR in the diagnosis and management of BPH clinical progression and LUTS.

## Supplementary Information

Below is the link to the electronic supplementary material.

Supplementary file1 (DOCX 412 KB)

## Data Availability

No datasets were generated or analysed during the current study.
