# Supplementary material for: The predictive value of prostate spherical volume ratio in lower urinary tract symptoms and clinical progression of benign prostatic hyperplasia: a retrospective cohort study
Source: Int Urol Nephrol. 2025 Jan 15;57(7):2023–32. doi: 10.1007/s11255-024-04355-4 (PMC12167308; doi:10.1007/s11255-024-04355-4)

**Figure S1:** Correlation between IPSS and, PV, IPP, PUL,PCAR, PSVR; IPSS=International prostate symptom score; PV=Prostate Volume; IPP=intravesical prostatic protrusion; PUL=prostatic urethral length; PCAR=presumed circle area ratio; PSVR=prostate spherical volume ratio


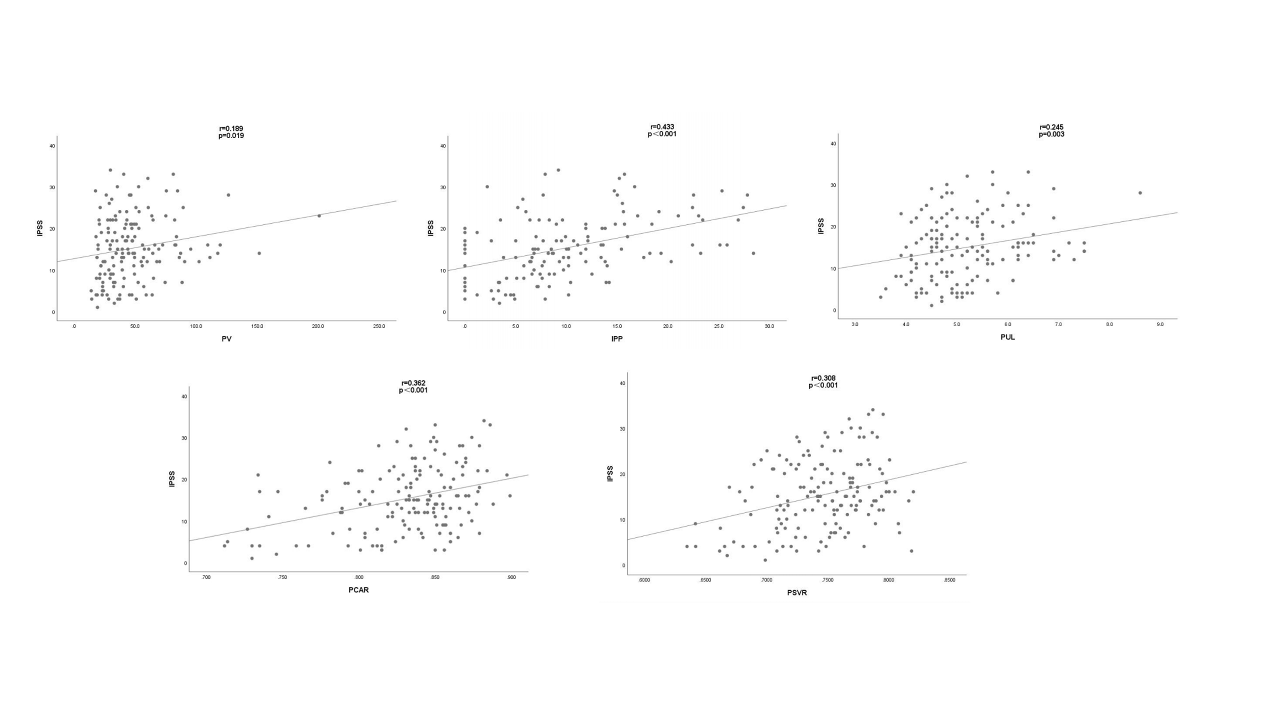


**Figure S2:** Correlation between Qmax and PV,IPP,PUL,PCAR, PSVR. Qmax=Maximum flow rate ;BMI=body mass index ;PV=Prostate Volume;IPP=intravesical prostatic protrusion;PUL=prostatic urethral length;PCAR=presumed circle area ratio; PSVR=prostate spherical volume ratio


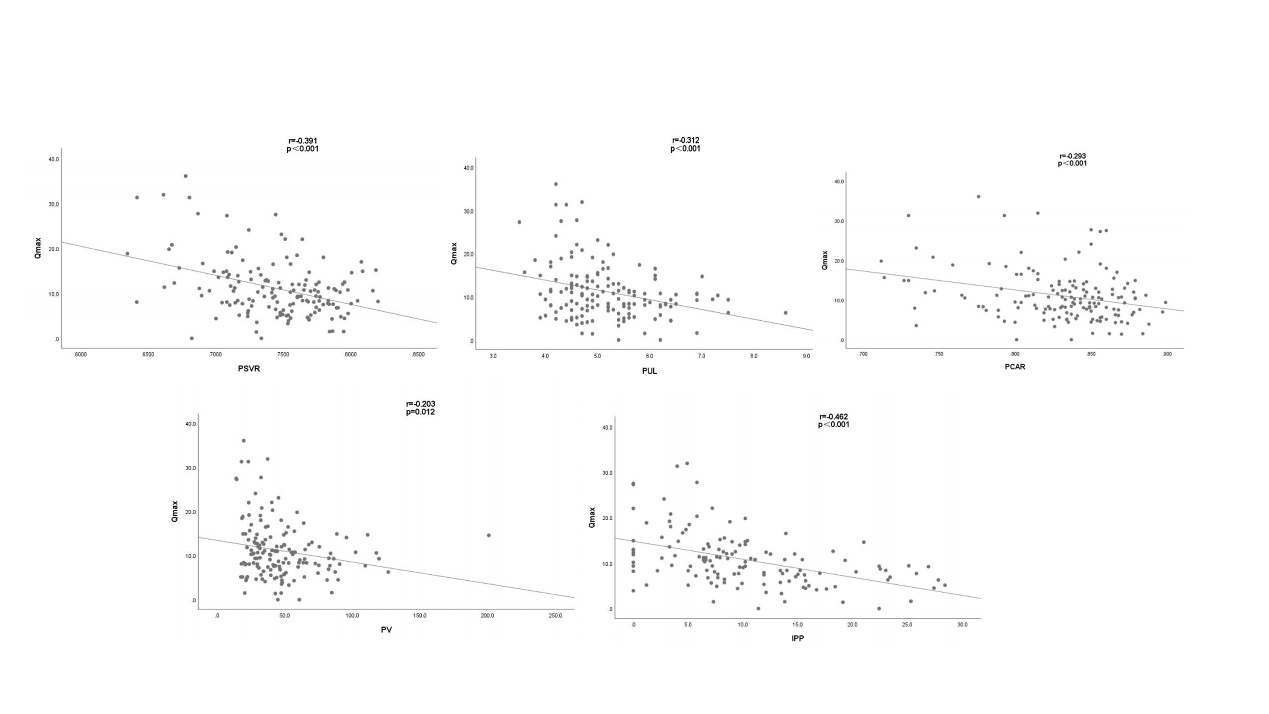

Supplement: Supplementary file 1 — Supplementary file1 (DOCX 412 KB) [file 11255_2024_4355_MOESM1_ESM.docx]
